# Three main metabolites from *Wolfiporia cocos* (F. A. Wolf) Ryvarden & Gilb regulate the gut microbiota in mice: A comparative study using microbiome-metabolomics

**DOI:** 10.3389/fphar.2022.911140

**Published:** 2022-08-03

**Authors:** Yong Lai, Hailun Yu, Huiling Deng, Qi Fang, Hui Lei, Li Liu, Nannan Wu, Xiurong Guo, Can Song

**Affiliations:** ^1^ School of Pharmacy, Southwest Medical University, Luzhou, Sichuan, China; ^2^ Key Laboratory of Condiment Supervision Technology for State Market Regulation, Chongqing Institute for Food and Drug Administration, Chongqing, China

**Keywords:** *Poria cocos*, polysaccharides, gut microbiota, microbiome, metabolomics

## Abstract

*Wolfiporia cocos* (F. A. Wolf) Ryvarden & Gilb, also known as *Poria cocos* is an ancient edible and medicinal mushroom that has been valued for thousands of years for its tranquilizing, diuretic, and spleen-enhancing properties. Because of the mushroom’s complex composition, its pharmacological effects have not been fully clarified. Therefore, to expand our knowledge of these effects from a pharmacological perspective and exploit potential medicinal value of fungal mushroom, we extracted three main metabolites from *P. cocos*, including water-soluble polysaccharides (PCX), water-insoluble polysaccharides (PCY), and triterpenoid saponins (PCZ) for intragastric injection into mice. These injections were made to explore the component’s effects on the mice’s gut microbiota and their metabolomics. The microbiota analysis showed that PCY had the strongest effect on regulating gut microbiota through altering its composition and increasing the number of *Lactobacillus* (*p* < 0.01). A total of 1,828 metabolites were detected using metabolomics methods, and the results showed that the three main active metabolites of *P. cocos* significantly changed the content of short-chain peptides in intestinal metabolites. In conclusion, our study further investigated the pharmacological functions of *P. cocos*, and revealed the differing effects of its three main metabolites on gut microbiota. The results suggested that PCY is a prominent prebiotic, and provided us with new insights into the potential development of fungal polysaccharides in Chinese traditional medicine.

## Introduction

Accumulated evidence indicates that natural products and their bioactive components, which are macromolecular bioactive carbohydrates with several biological activities and a high potential application value in the pharmaceutical field, have attracted considerable attention owing to their diverse bioactivities (Miaoyu Li et al., 2021). A series of clinical studies have demonstrated that polysaccharides from fungi possess antitumor, immunomodulatory, anti-inflammatory, antioxidant, anti-aging, anti-hepatitis, and anti-diabetes properties (Hu et al., 2017; Yang et al., 2021; Yu et al., 2021). Research on gut microbiota has become increasing prevalent in recent years; animal guts contain a remarkable abundance of microorganisms that are intimately related and play a major role in maintaining overall health (Pickard et al., 2017). A healthy gut microbial community creates a natural protective barrier for the body and ensures proper immune functions, inflammatory signaling, nutrient delivery, and beneficial metabolism of short-chain fatty acids, choline, bile acids, and lipids (Schroeder and Backhed, 2016; Cheung et al., 2020). In addition, the gut microbiota is also known as the “second genome” of the body (ChaominYin et al., 2020). Recent studies have gradually revealed the relationship between the gut microbiota and different organs of the body, from the gut-brain axis, gut-liver axis to gut-skin axis, and the functions of gut microbes are being uncovered (Cryan et al., 2019). The interaction between gut microbiota and its host regulatory systems is widely explored in a variety of neurodegenerative diseases and metabolic diseases, thus demonstrating the balance of gut microbes are important for human health (Gomes et al., 2018; Morais et al., 2021).

*Wolfiporia cocos* (F. A. Wolf) Ryvarden & Gilb, also known as *Poria cocos*, an ancient traditional Chinese medicine, is an edible and pharmaceutical mushroom belonging to the dry sclerotium of Polypo-raceae fungi widely used in many Asian countries (Sun, 2014). Named Fu-ling in Chinese, *P. cocos* is a highly valued tonic mushroom that grows around the old or dead roots of pine trees (Cheng et al., 2021). The sclerotium of *P. cocos* has been used to treat edema, nephrosis, spleen deficiency, chronic gastritis, acute gastroenteric symptoms, diarrhea, indigestion, and weight loss for more than two thousand years, and it can also be consumed by healthy people to calm the nerves and strengthen the body’s immune function (Ji et al., 2019; Li et al., 2019; Weifeng Li et al., 2021). This mushroom is a high-grade healthy food that widely used to strengthen the spleen and immune system in Chinese traditional medicine and food culture; moreover, it appears to have beneficial effects on absorption and metabolism (Kim et al., 2019; Junsheng Liu et al., 2021). Modern medicine has shown that *P. cocos* contains polysaccharides, triterpenes, fatty acids, ergosterol gum, and chitin, etc. of which water-soluble polysaccharides (PCX), water-insoluble polysaccharides (PCY), and triterpenoid saponins (PCZ) are the three main metabolites (Feng et al., 2019). In recent years, a growing body of research has shown that the PCX of *P. cocos* has a regulatory effect on gut microbiota (Zhu et al., 2020). For example, polysaccharides from *P. cocos* can reduce inflammatory factors and blood lipid levels to prevent atherosclerosis (Weifeng Li et al., 2021). *P. cocos* polysaccharides can also enhance immune system activity against lung cancer (Tian et al., 2019). Research reports on the effects of water-insoluble polysaccharides and triterpene saponins on gut microbiota have also been published in the last 2 years. These reports have also noted that PCY from the sclerotium of *P. cocos* can modulate hyperglycemia and hyperlipidemia by regulating intestinal microbiota (Chao et al., 2021). Triterpene saponins extracted from *P. cocos* can enhance non-specific immunity by activating the immune response of NK cells (Sun et al., 2019). To date, few experimental designs have been developed that utilize PCX, PCY, and PCZ to modify and compare gut microbes. To this end, the effects of the three metabolites of *P. cocos* on gut microbiota need to be urgently explored and investigated.

Numerous researches have demonstrated that the use of integrated approaches based on 16Sr RNA high-throughput sequencing and metabolomics to study the effects of polysaccharides on the gut microbiota has great potential (Manor et al., 2020). High-throughput sequencing and bioinformatics analysis can more effectively detect the changes of microbial diversity and species richness (Luo et al., 2022a). Non-targeted metabolomics is a research method that quantitatively analyzes main metabolites in organisms, and looks for the relative relationship between metabolites and physiological changes (Aon et al., 2020). Therefore, using an omics technology in combination with animal models is feasible and scientifically valuable to investigate the impact of the three major active metabolites from *P. cocos* on the gut microbiota of mice.

In this study, we extracted the three major active metabolites from *P. cocos* and orally administered them to healthy mice, and then investigated the pharmacological effects of these metabolites on the mice gut microbiota using 16Sr RNA high-throughput sequencing (HTS) and metabolomics analyses. We detail the interactions between PCX, PCY, PCZ, and gut microbiota, explore their effects on intestinal metabolites, and evaluate their potential ability to regulate the gut microbiota.

## Material and methods

### Chemicals and reagents

*P. cocos* (Schw.) Wolf was purchased from Sichuan Xin Ren Tai Pharmaceutical Co., Ltd. China. Serum and tissue levels of inflammatory cytokines [tumor necrosis factor alpha (TNF-α), interleukin 10 (IL-10), and gamma-interferon (IFN-γ)] were measured using commercial assays purchased from Beijing Sola Biotechnology Co. (Beijing, China). All solutions were prepared using double-distilled water, and the other chemicals were of analytical grade.

### Extraction and purification of PCX, PCY, and PCZ

**FIGURE 1:**
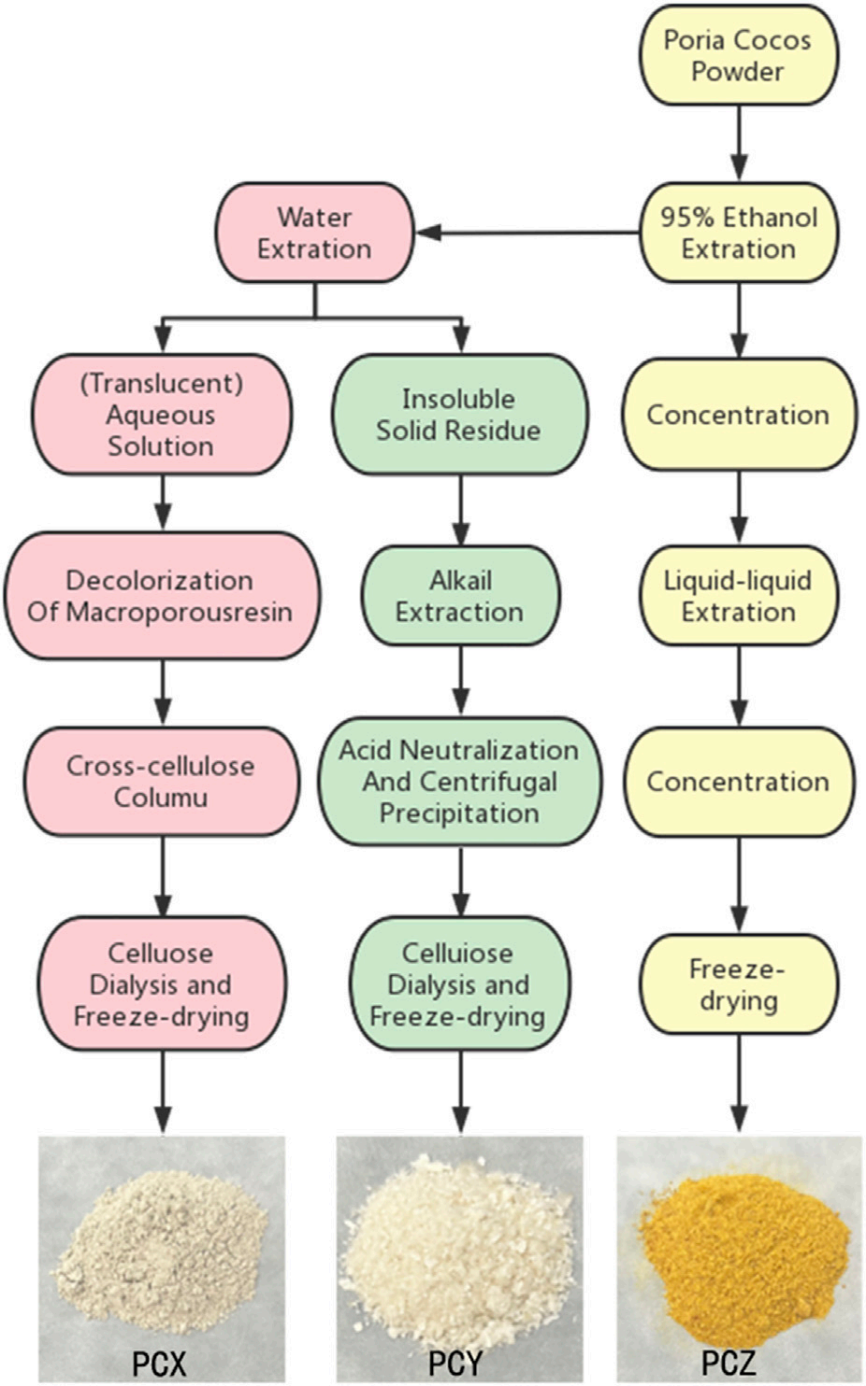
Schemes of extraction of three prominent active component fractions from *P. cocos.*

The extraction and purification methods for PCX, PCY, and PCZ were based on those of previous studies (RuiDian et al., 2010; Baosong Chen et al., 2019; Sun et al., 2019). As shown in Figure 1, PCX was extracted at a time of 6 h and a temperature of 80°C, and the extracted polysaccharide aqueous solution was precipitated in alcohol and decolorized. Purified PCX was then obtained using a macroporous adsorbent resin and ion-exchange cellulose column elution, followed by dialysis and freeze-drying. PCY was obtained by soaking the residue of *P. cocos* raw material following water extraction with NaOH (0.5 mol for 4 h). This operation was repeated three times to obtain a crude polysaccharide alkali solution, and then 0.5 mol hydrochloric acid was added to neutralize the alkali solution and thereby precipitate the polysaccharide. Following, centrifugation and washing with deionized water, pure PCY was obtained after removing impurities such as NaCl *via* dialysis using a cellulose dialysis bag. PCZ was obtained by soaking *P. cocos* powder in 95% ethanol; the solution was extracted 12 h five times, concentrated until no alcohol smell could be observed, and then extracted with petroleum ether five times, with the petroleum ether layer being discarded, and the aqueous layer being retained. The remaining aqueous layer was then extracted with ethyl acetate five times after the polar molecular compounds were obtained. All extracts from *P. cocos* were dried and stored at −20°C for subsequent experiments.

### Characterization of component fractions from *P. cocos*

Referring to the previous studies and established methods (Xu et al., 2019), the total sugar content of PCY was determined using the sulfuric acid-phenol method with glucose as a control. Then, the absorbance was detected using UV-visible spectrophotometer at 490 nm, and the total sugar content was calculated by drawing a standard curve. The monosaccharide components of polysaccharides were determined by ion chromatography. A Thermo ics5000+ ion chromatography system ics5000+, (Thermo Fisher Scientific, United States) and Dionex ™ CarboPac ™ PA10 (250 * 4.0 mm, 10 um) liquid chromatographic column was utilized, with an injection volume of 10 uL. Mobile phase A was H_2_O, mobile phase B 100 mM NaOH, the column temperature was 30°C, and the monosaccharide components were analyzed and detected using an electrochemical detector.

### Animal experiment

Thirty-two male Kunming (KM) mice (18–22 g, 4–6 weeks old) were obtained from the Animal Experiment Center of Southwest Medical University (Luzhou City, Sichuan Province, China). All animal care and procedures strictly followed the guidelines approved by the Animal Care and Use Committee of the Southwest Medical University (permit number 20211207-005). The mice were housed in individually ventilated cages under controlled humidity (60%) and temperature (23°C), with 12 h of light and 12 h of darkness. Each mouse had free access to food and water, and their weights and food consumption rates were monitored and recorded daily. After 5 days of growth and adaptation to the environment, the mice were randomly divided into PCX, PCY, PCZ, and the control (CNT) groups. Every morning, the PCX group (300 mg/kg/day, intragastric water-soluble polysaccharide administration for 15 days) (Cheng et al., 2021), PCY group (300 mg/kg/day, intragastric water-insoluble polysaccharide administration for 15 days) (Sun et al., 2019), PCZ group (150 mg/kg/day, daily intragastric triterpene saponin administration for 15 days) (Chao et al., 2021), CNT group (with an equal amount of sterile distilled water by intraperitoneal injection, once daily for 15 consecutive days). At the end of the experiment, the male KM mice were fixed on the console, and their anuses were gently wiped with sterile cotton swabs to initiate a stress response and cause the mice to defecate. Mouse feces were collected and placed in sterilized test tubes, and microbial composition was evaluated in mouse fecal samples using 16S rRNA HTS and metabolomic analysis. The liver, spleen, and blood of mice were collected for ELISA determination of the inflammatory cytokines (TNF-α, IL-10, and IFN-γ). All samples were transferred into liquid nitrogen, and stored in a freezer (−80°C) for future research and analysis.

### 16Sr RNA high-throughput sequencing and bioinformatics analysis

A genomic DNA isolation kit was used to extract DNA from mouse feces (Song et al., 2022); the 16S rRNA gene was amplified from genomic DNA *via* a polymerase chain reaction (PCR), with the universal bacterial primers 16s-F (5′-AGAGTTTGATYMTGGCTCAG-3) and 16s-R (5-TGCTGCCTCCCG PCZGGAGT-3′) targeting the hyper-variable V3–V4 region of the 16S rRNA gene of bacteria. This gene was then subjected to HTS, which was performed at the Majorbio Bio-Pharm Technology Co., Ltd. (Shanghai, China). Prior to the analysis, the sequences were demultiplexed and quality-filtered using the QIIME2 platform. Following quality control, the sequences were analyzed using QIIME2 (v2020.2) and the sequences with a 100% similarity were classified as an amplicon sequence variants (ASV). The 16Sr DNA genes were assigned to different classification categories using the ribosomal database item classifier algorithm and Silva (SSU123) database, and differences between the two groups were tested using the Wilcoxon rank-sum test. The component differences between the two groups were analyzed using principal coordinate analysis (PCoA). The linear discriminant analysis effect size (LEfSe) was used to determine the reasons for the differences in bacterial composition and the selected differences were sorted using a linear discriminant analysis (LDA) > 2.0.

### Sample preparation and LC–MS analysis for metabolomics

Non-targeted metabolomic liquid chromatography mass spectrometry (LC-MS), in combination with multivariate statistical analysis, was carried out to explore fecal metabolic changes in mice after the administration of the three prominent active substances from *P. cocos* (Chen et al., 2017; Dan-Qian Chen et al., 2019). The entire method flow included metabolomic procedures, including sample preparation, metabolite separation and detection, data pre-processing, and statistical analysis for metabolite identification, which was performed at the Majorbio Bio-Pharm Technology Co., Ltd. (Shanghai, China). In our study, we used the Kyoto Encyclopedia of Genes and Genomes (KEGG), Venn diagram, Partial least squares Discriminant Analysis (PLS-DA), and correlation analysis between metabolic and families to directly visualize the differences in metabolic profiles of the four groups and detect metabolites that distinguished the different groups. Thereafter, we carried out pairwise comparisons using the Wilcoxon rank-sum test and Kruskal-Wallis H test to evaluate metabolite abundance differences among the four groups.

### Enzyme-linked immunosorbent assay

An enzyme-linked immunosorbent assay (ELIAS) refers to a qualitative and quantitative assay that binds soluble antigens or antibodies to a solid-phase carrier such as polystyrene and uses the specific binding of antigens and antibodies for the immune reaction. Mouse liver and spleen tissues were washed with saline, added to a PBS solution (pH 7.2–7.4) that was subjected to ultrasonic trituration, and finally centrifuged at 5,000 rpm for 10 min to obtain a homogenate of liver and spleen tissues. The levels of inflammatory cytokines tumor necrosis factor alpha (TNF-α), interleukin 10 (IL-10), and interferon gamma (IFN-γ) in the serum and tissue were determined using a mouse ELISA kit (Beijing Solarbio Science Co., Ltd. Beijing, China) according to the manufacturer’s instructions. The activities of TNF-α, IFN-γ, and IL-10 were expressed as pg/ml, the absorbance of all samples and standard reaction wells was measured at 450 nm on a microplate reader (Molecular Devices, Sunnyvale, CA, United States), and the concentration of cytokines was calculated according to the standard curve.

### Statistical analysis

Data from all analyses were expressed with the mean ± standard deviation (SD). We evaluated and calculated the significance of the differences between the groups using the statistical software GraphPad Prism (7.04). The significance of differences between two groups was tested using the Student’s unpaired *t*-test. Comparisons between multiple groups were performed using one-way ANOVA followed by Dunnett’s post-hoc test. The abundance of gut microbial bacteria and their metabolites was determined using nonparametric tests, including the Wilcoxon rank-sum test and Mann-Whitney *U* test. Fecal metabolite intensities were tested for association with 16S ribosomal RNA levels using Spearman’s rank correlation. Metabolomic data were subjected to PLS-DA using SIMCA 14.0 (Umetrics, Sweden) to construct multivariate statistical models. The significant differences in ELISA data were tested using the independent *t*-test. Values of *p < 0.05, **p < 0.01, and ***p < 0.001 were considered statistically significant for data analyses.

## Results

### General data of mice

After 15 days of feeding under suitable conditions, the relative body weights of the mice in the PCX, PCY, and PCZ groups were significantly reduced compared to those in the blank control group (Supplementary Figure S1). As shown in Supplementary Figure S2, the average daily food consumption rates of mice in the PCX, PCY, and PCZ groups were reduced compared to that of the blank control group. These results suggest that the orally administration of *P. cocos* reduced the diet of mice and facilitated weight loss.

### Characterization of polysaccharides

The total sugar content was determined using the sulfuric acid-phenol method. The total sugar content was of PCX calculated as 80.48%. The monosaccharide components of polysaccharides were determined by ion chromatography. PCX was composed of xylose, mannose, glucose, and galactose, and PCY was mainly composed of glucose in this study (Supplementary Table S1).

### Effects on the composition and structure of gut microbiota

We performed high-throughput gene sequencing of 16Sr RNA in the fecal bacterial DNA of four types of mice, and the sequencing data adequately reflected the richness and evenness of the microbial community in each sample. A total of 8,26,747 high quality reads (>200 bp) were obtained from 32 stool samples for taxonomic identification and diversity analyses. The Ace, Shannon, Simpson, Shannoneven, Simpsoneven, and coverage diversity indices for the four groups are shown in Supplementary Table S2, and the sparsity curves used to examine the richness of the sequencing depth (Sobs index) and the number of shared ASVs are shown in Supplementary Figure S3. Rarefaction curves can be used to compare species diversity in samples with different sequencing data bulk, and they can also be used to explain whether the sequencing data bulk of the samples is reasonable. Most of the samples reached plateau levels, which indicated that the sequencing depth for detecting the gut microbiome genes was adequate. The ASV average values of the PCX (303.3) and PCZ (297.3) was higher than that of the CNT (288.1); however, the ASVs were lower in mice treated with PCY (246.5), and all differences were not statistically significant.

**FIGURE 2:**
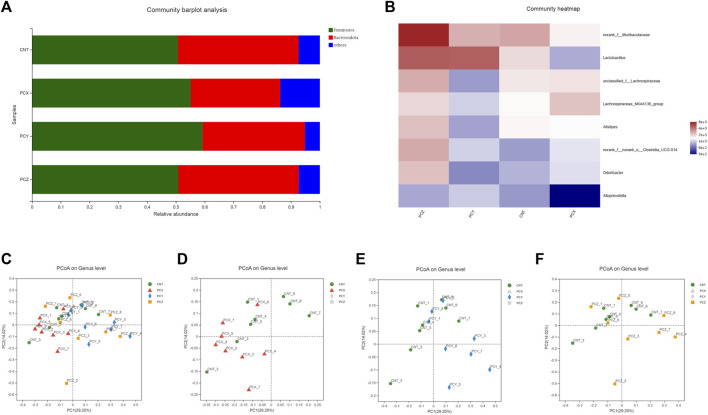
**(A)** Bacterial taxonomic distributions in 16S rDNA sequencing data from mouse feces at the phylum level. **(B)** Heatmap of fecal microbiota at the genus level. **(C)** Score scatter plots of the PCoA classification of fecal microbiota of four groups together. **(D)** Score scatter plots of the PCoA classification of fecal microbiota in the PCX and CNT group. **(E)** Score scatter plots of the PCoA classification of fecal microbiota in the PCY and CNT group. **(F)** Score scatter plots of the PCoA classification of fecal microbiota in the PCZ and CNT group.

To obtain a clear picture of the microorganisms contained in each group and the relative abundance of each microorganism, we used a community bar plot analysis to observe the structure of the gut microbial community at the phylum level in the four groups. As shown in Figure 2A, *Firmicutes*, *Bacteroides, Campylobacterota, Patescibacteria, Actinobacteriota, Desulfobacterota* and *Deferribacterota* were the main bacterial phyla present in the intestinal flora of mice. *Firmicutes* was the most dominant intestinal bacterial community in the experimental and control groups, and *Bacteroides* was the second most dominant intestinal bacterial community. We created a heatmap of the structural distribution of the intestinal microbiota to determine differences in the intestinal community at the genus level in mice gavaged with PCX, PCY, and PCZ (Figure 2B). This map visually represents the magnitude of the data values in defined shades of color, with higher abundances being darker and lower abundances being lighter. Compared with the CNT group, PCY altered the genus level of the intestinal microbiota in mice, with the most abundant bacteria being *Lactobacillus,* norank-f-Muribaculaceae, Alloprevotella, and unclassfied-f-Lachnospiraceae.

To determine the potential effects of the three metabolites of *P. cocos* on the healthy intestines of mice, we used PCoA to visualize the divergency in gut microbial community structure and diversity between groups. The PCoA plot of the four groups were mainly clustered in the upper part of the region, with PC1 accounting for 29.20% and PC2 accounting for 14.02%. As shown in Figure 2C, the PCX group is mainly clustered in the left side of the plots, and the PCY group is clustered in the right side of the plots, without any intersection between the two groups; by contrast, the PCZ and CNT groups are scattered and not clustered in specific areas. Compared with the CNT group on the graph, the PCX and CNT groups were in two different regions on the left and right in Figure 2D; excitingly, the PCY group was clearly clustered in a region far away from the CNT group (Figure 2E), but the PCZ and CNT were interlaced in the general area with each other and were not separated (Figure 2F). In general, the results indicated that the variation in the structure of the gut microbiota was closely related to the administration of *P. cocos* polysaccharides.

### Comparative analysis of gut microbiota

**FIGURE 3:**
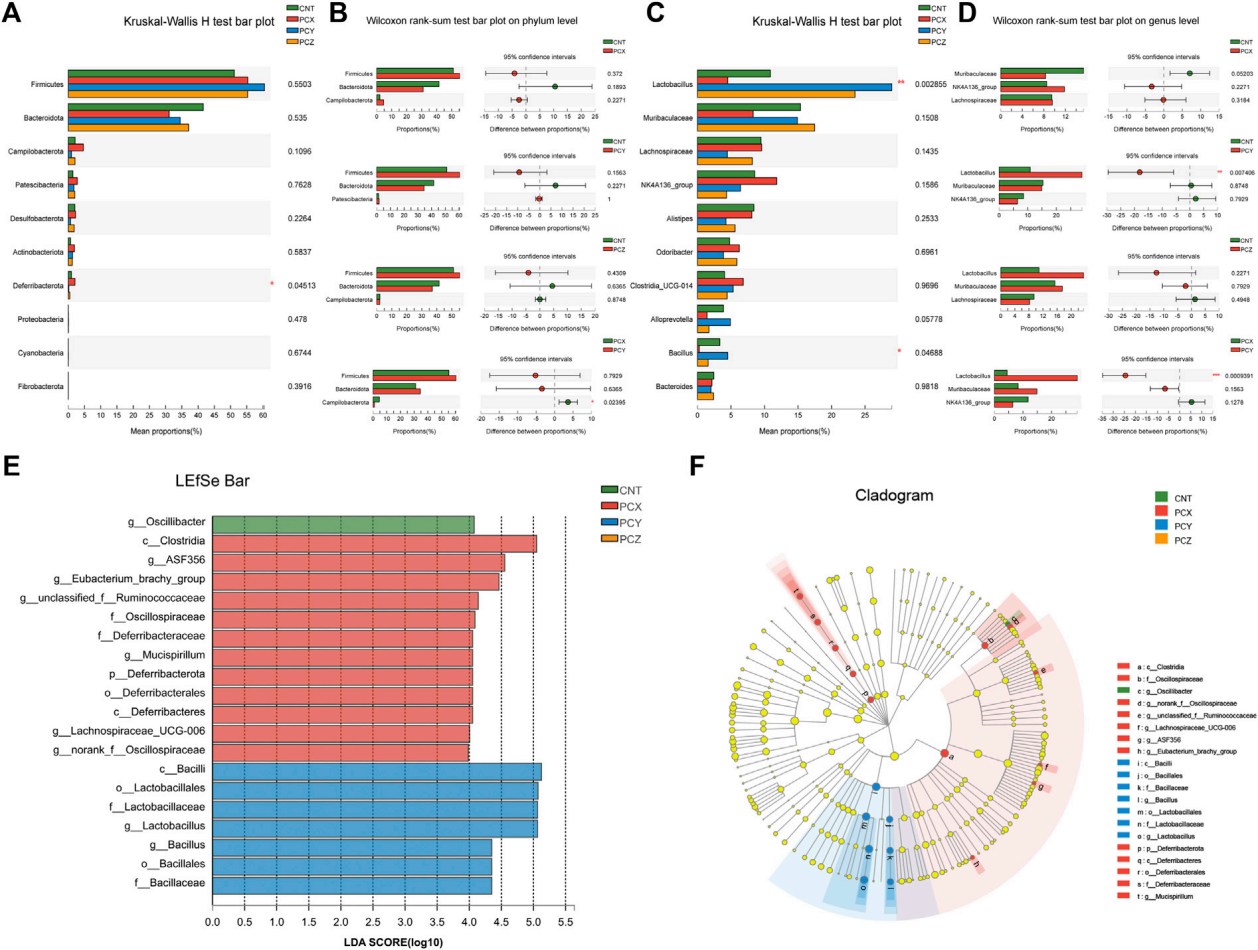
**(A)** Kruskal-Wallis H test bar plot of fecal microbiota at the phylum level. **(B)** Wilcoxon rank-sum test bar plot of fecal microbiota at the phylum level between the two groups. **(C)** Kruskal-Wallis H test bar plot of fecal microbiota at the genus level. **(D)** Wilcoxon rank-sum test bar plot of fecal microbiota at the genus level between the two groups. **(E)** The LDA scores of four groups were obtained by LEfSe analysis. **(F)** Taxonomic cladogram generated from LEfSe analysis of 16S rRNA gene sequences.

Modifications of the gut microbial community in mice were quantitatively determined at the phylum level using 16S rRNA HTS; phyla whose relative abundance was <1% were excluded. The top 10 phyla with high relative abundances were selected for comparison, as shown in Figure 3A. The gut microbial taxa of the four groups consisted mainly of *Firmicutes*, *Bacteroides,* and *Campilobacterota* at the phylum level, and the increase of *Deferribacterota* in the PCX group compared to other three groups was statistically significant (*p* < 0.05). Apparently, the comparison between the two groups indicated that the ratio of *Firmicutes* to *Bacteroides* increased following the administration of the *P. cocos* active metabolites (Figure 3B).

To further investigate the effects of the three metabolites on intestinal microorganisms at the genus level in the four groups, we selected the top 10 species in relative abundance to perform the Kruskal-Wallis H test; the results are shown in Figure 3C. PCY-treated mice showed a significant increase in the abundance of *Lactobacillus* (*p* < 0.01) compared to the other three groups. In addition, the abundance of *Bacillus* was significantly attenuated following administration of water-soluble polysaccharides in PCX group. Moreover, we also created Wilcoxon rank-sum bar plots at the genus level to determine divergences between the two groups (Figure 3D). PCX increased the number of Lachnospiraceae and decreased the number of Muribaculaceae compared with the CNT group*.* PCZ increased the number of *Lactobacillus* and Muribaculaceae compared with the CNT group, but none of these results were statistically significant, and only the PCY group showed a significant increase in the number of *Lactobacillus* (*p* < 0.01).

The linear discriminant analysis effect size (LEfSe) analysis is a method for discovering and interpreting high-latitude data markers to determine features that best explain species-to-species differences and the effects of these features. In this study, we compared high-dimensional categories, and detected a difference in the dominance of bacterial communities among the four groups. Based on the effects of each group of bacterial genera with a preset value of two, the higher the linear discriminant analysis (LDA) score, the more significant it was in the comparison, and conversely the lower the score the less significant it was. As shown in Figures 3E,F, the LEfSe analysis of gut microbial community demonstrated that 20 species contributed to the difference in relative abundance among the four groups, with PCX contributing 12 species, PCY seven species, and CNT one species. The predominant bacteria that caused the variation in the gut microbiota were *Lactobacillus, Clostridia, Alistipes, Odoribacter,* Lachnospiraceae_NK4A136_group*,* and Lachnospiraceae. In addition, the results of the LEfSe Bar analysis between the experimental groups and the blank control group are shown in Supplementary Figures S4–S6.

### Effects on fecal metabolomics

**FIGURE 4:**
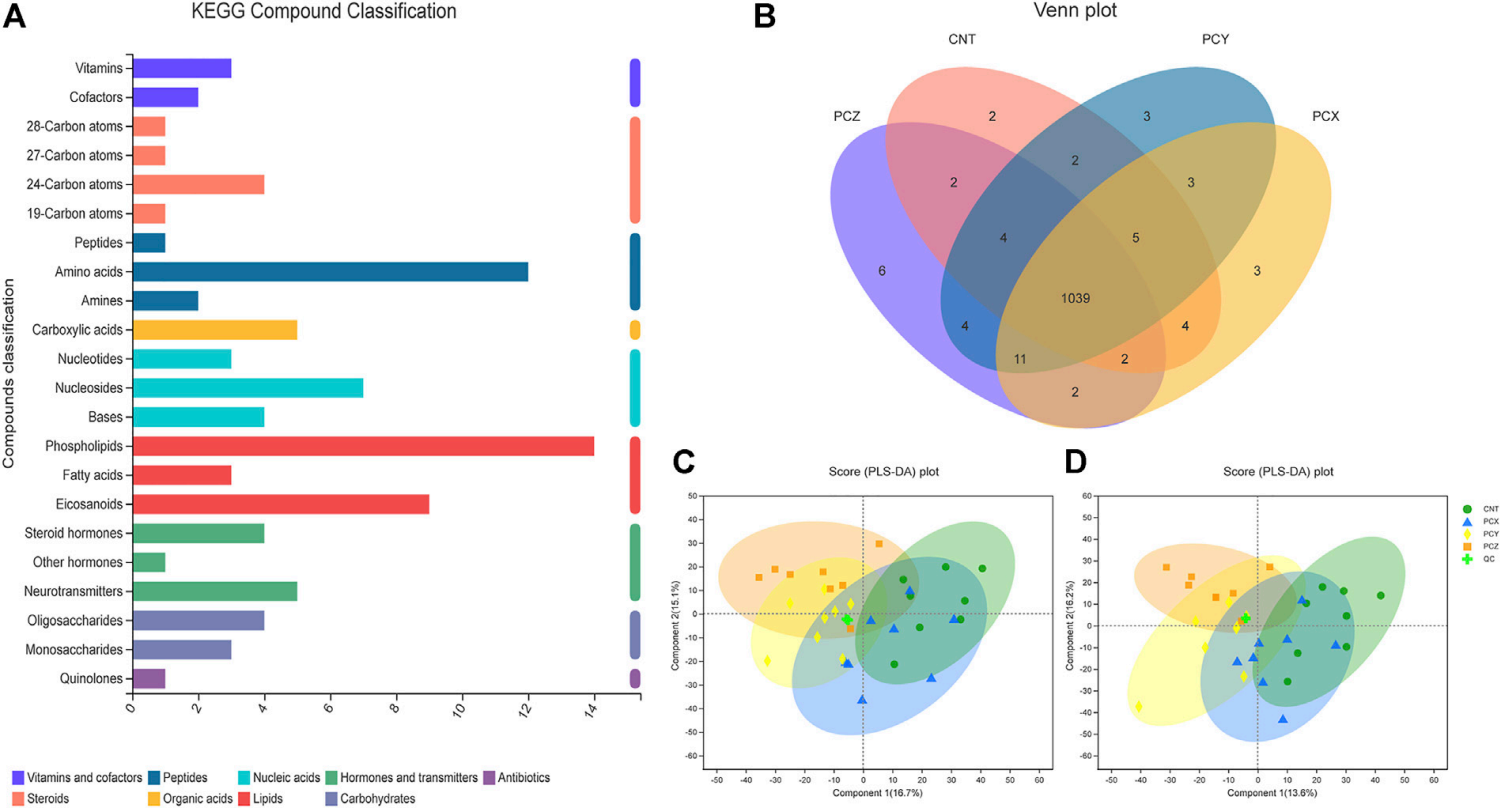
**(A)** KEGG compound classification of metabolites from four groups of mice. **(B)** Venn diagram of metabolites from four groups of mice. **(C)** PLS-DA score plots of metabolic profiling of fecal samples in positive ion mode. **(D)** PLS-DA score plots of metabolic profiling of fecal samples in negative ion mode.

A total of 1,828 metabolites were detected using metabolomics methods. To identify the potential effects of different component fractions on healthy mice, we incorporated KEGG, a Venn diagram, and PLS-DA to directly visualize the four groups’ discrepancies in metabolic profiles and detect metabolites that distinguished the different groups (Figure 4). KEGG compound classification is a method of classifying metabolites according to the hierarchical level of biological functions in which they are involved. We compared metabolites from four groups of mice to the KEGG compound database to obtain a metabolite classification profile, as shown in Figure 4A; 22 types of substances occupy a dominant position in the intestinal metabolic profile, including vitamins, cofactors, steroids, peptides, organic acids, nucleic acids, lipids, carbohydrates, antibiotics, hormones, and transmitters.

As shown in the Venn diagram (Figure 4B), a total of 1,039 metabolites overlap among the four groups, thereby indicating that these substances are common to them; individually, the CNT group has only two unique metabolites, the PCZ group has six unique metabolites, and the PCX and PCY groups each have three unique metabolites.

PLS-DA scoring plots in both positive and negative ion modes showed that after the mice were treated with different component fractions, there was a clear classification of microbiota composition across groups (Figures 4C,D), with samples from the same group clustered together. The percentage changes in the first two components in negative ion mode were 16.7 and 15.1%, and in the positive ion mode they were 13.6 and 16.2%, respectively. The PCY and PCX groups were clearly clustered together, and the PCX group was closest to the CNT group, whereas PCY and PCZ were further away from the CNT group. The results indicated that PCY caused a greater alteration in the metabolic composition of mouse feces.

### Comparative analysis of metabolomics

**FIGURE 5:**
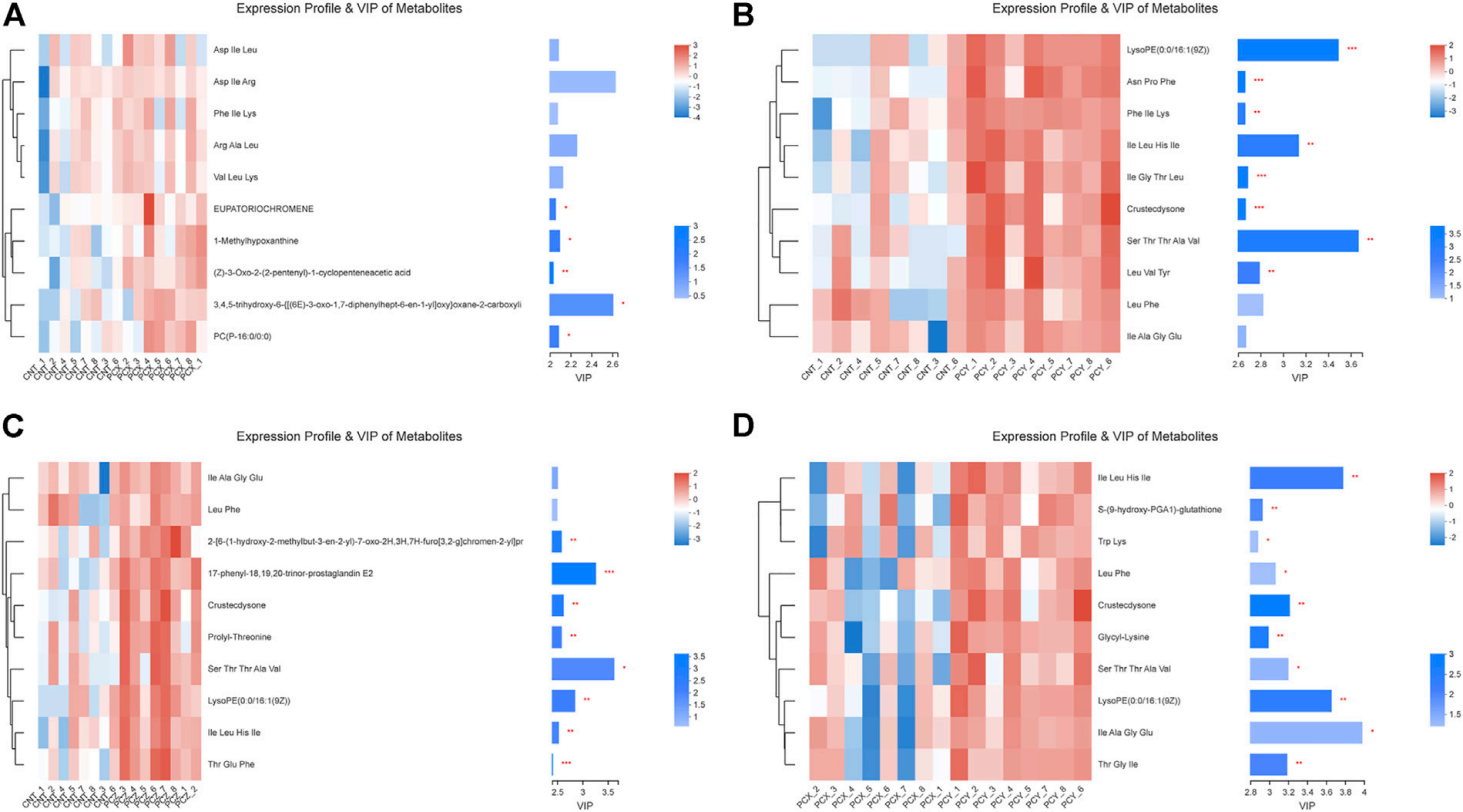
**(A)** Expression profile and VIP of metabolites between the PCX and CNT groups. **(B)** Expression profile and VIP of metabolites between the PCY and CNT groups. **(C)** Expression profile and VIP of metabolites between the PCZ and CNT groups. **(D)** Expression profile and VIP of metabolites between the PCX and PCY groups. Differences were considered statistically significant at *p < 0.05; **p < 0.01; ***p < 0.001 level.

Variable important in projection (VIP) analysis was performed using a clustering heat map and VIP bar graph to detail the expression pattern of metabolites in the four groups and the *p* value of metabolites in a multivariate statistical analysis of VIP and unidimensional data; this approach visualizes the importance and expression trend changes in differential metabolites. As shown in Figures 5A–C, these metabolites are mainly involved in amino acid metabolism, fatty acid metabolism, and purine metabolism, respectively, with short peptide metabolites accounting for the majority of the top ten metabolites in each group. Both PCY and PCZ significantly increased the contents of LysoPE 0:0/16:1 (9Z), Ile Leu His Ile, Crustecdysone, and Ser Thr Thr Ala Val (*p* < 0.05). And PCX significantly increased the contents of EUPATORIOCHROMENE, 1-Methylhypoxanthine, (Z)-3-Oxo-2-(2-pentenyl)-1-cyclopenteneacetic acid, 3,4,5-trihydroxy-6-{[(6E)-3-oxo-1,7-diphenylhept-6-en-1-yl]oxy}oxane-2-carboxyli, and PC(P-16:0/0:0) (*p* < 0.05). In addition, score plots of the VIP heatmap (Figure 5D) reveal the separation between the PCY and PCX groups on the level of metabolites, with a total of nine differential short-chain polypeptide metabolites identified.

### Correlation analysis between microbiome and metabolomics

**FIGURE 6:**
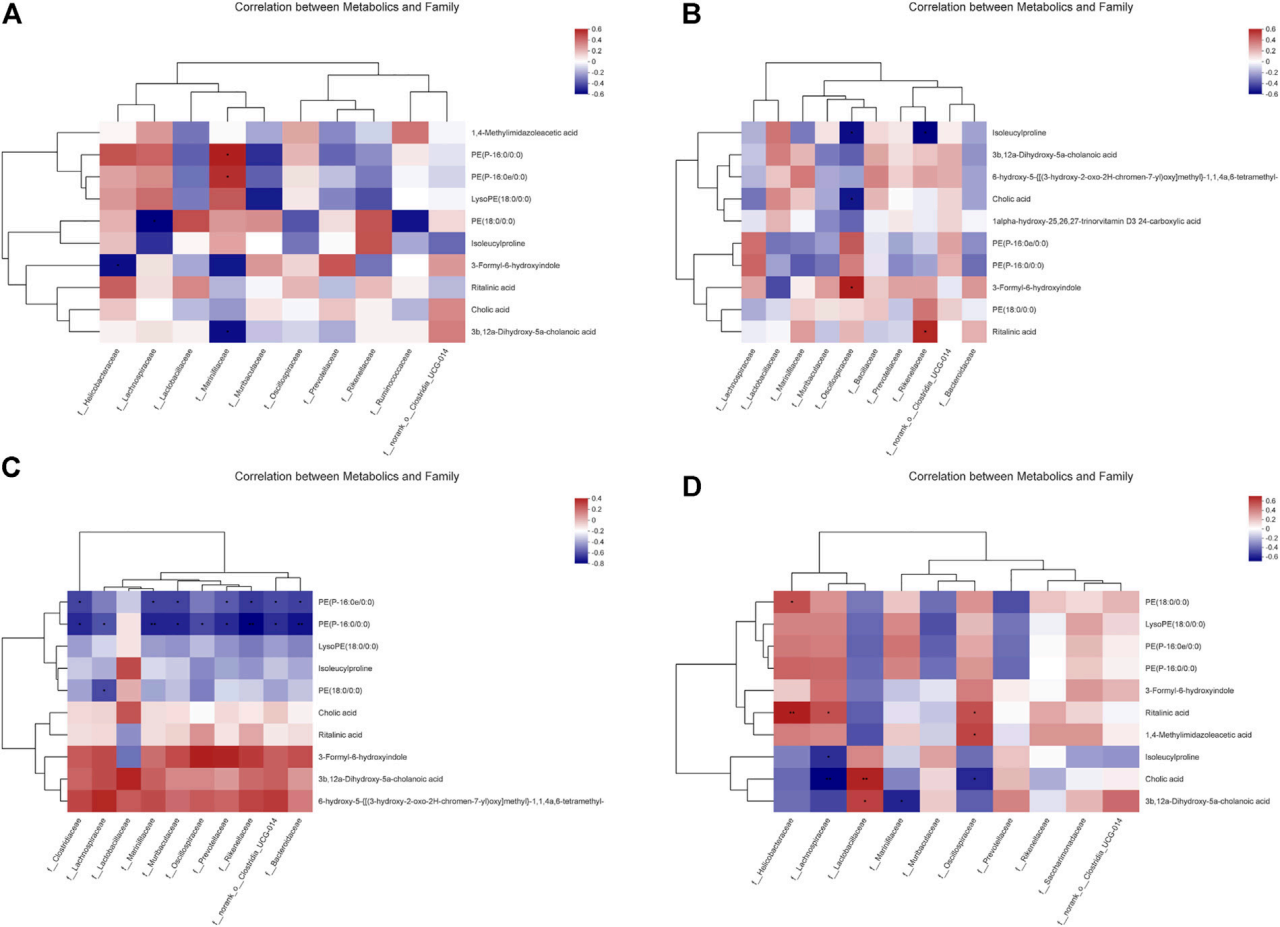
**(A)** Correlation between metabolics and family between the PCX and CNT group. **(B)** Correlation between metabolics and family between the PCY and CNT group. **(C)** Correlation between metabolics and family between the PCZ and CNT group. **(D)** Correlation between metabolics and family between the PCX and PCY group. A connection represents a strong Spearman’s correlation coefficient (ρ > 0.5) and significant (*p* value <0.05) correlation.

We further hypothesized that the active metabolites of *P. cocos* may interfere with the natural intestinal barrier through a network between bacteria and metabolites. Accordingly, we analyzed the correlation between microbiomes and metabolomics to search for possible alterations in bacterial and metabolite populations, and potentially reveal the relationship between bacterial components and complex metabolites. As shown in Figure 6, compared with the CNT group, Marinifilaceae increased PE (p-16:0e/0:0) and PE (p-16:0/0:0) and decreased the amount of 3b, 12a-dihydroxy-5a-cholanoic acid in the PCX group (Figure 6A). In PCY group (Figure 6B), Oscillospiraceae increased the production of 3-formyl-6-hydroxyindole and inhibited the production of isoleucylproline and cholic acid. Rikenellaceae also increased ritalinic acid, and isoleucylproline was reduced. Surprisingly, in the PCZ group (Figure 6C), almost all bacteria were positively correlated with the production of 3-formyl-6-hydroxyindole, 3b, 12a-dihydroxy-5a-cholanoic acid, and 6-hydroxy-5-{[(3-hydroxy-2-oxo-2h-chrome-7-yl) oxy] methyl}-1,1,4a, 6-tetramethyl -, and negatively correlated with the number of PE (p-16:0e/0:0), and PE (p-16:0/0:0). Both PCX and PCY groups (Figure 6D) comprised polysaccharides, and their comparison showed that Helicobacteraceae significantly elevated ritalinic acid and *Lactobacillaceae* significantly elevated cholic acid levels at a statistically significant level (*p* < 0.01).

### Effects of three metabolites on cytokines

**FIGURE 7:**
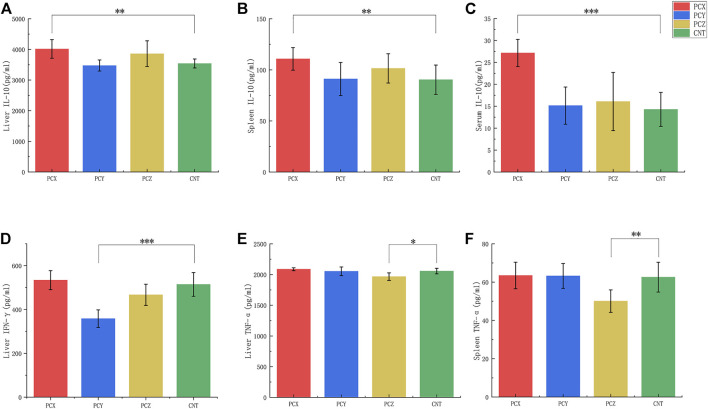
Levels of tumor necrosis factor-α (TNF-α), interleukin 10 (IL-10), and Interferon gamma (IFN-γ) in serum, liver, and spleen tissues. **(A)** Levels of IL-10 in liver tissues. **(B)** Levels of IL-10 in spleen tissues. **(C)** Levels of IL-10 in serum. **(D)** Levels of IFN-γ in liver tissues. **(E)** Levels of TNF-α in liver tissues. **(F)** Levels of TNF-α in spleen tissues. All values are mean ± SD, differences were considered statistically significant at *p < 0.05, **p < 0.01, ***p < 0.001 level.

The results of the ELISA are shown in Figure 7. Compared with the control group, PCX significantly elevated IL-10 levels in the liver tissues (*p* < 0.01, Figure 7A), spleen tissues (*p* < 0.01, Figure 7B), and serum (*p* < 0.001, Figure 7C) of mice**,** PCY significantly decreased IFN-γ levels (*p* < 0.001, Figure 7D) in the livers of mice, and PCZ decreased TNF-α levels in their liver (*p* < 0.05, Figure 7E) and spleen (*p* < 0.01, Figure 7F) tissues. PCX and PCZ also promoted the secretion of the interferon *γ* (IFN-γ) immune response and enhanced innate specificity. In addition, there was no significant difference in TNF-α and IFN-γ levels in response to PCX, and there was no significant difference in IL-10 levels in response to PCY and PCZ. Our results showed the active metabolites derived from *P. cocos* promoted the production of inflammatory cytokines to strengthen the spleen and enhance immunity, although the levels of some cytokines did not change.

## Discussion

A healthy gut relies on a balance of commensal bacteria, probiotics and pathogens, especially probiotics, which play an essential role in regulating the gut microbiota (Feng et al., 2018). Prebiotics, a class of organic substances that are not digested by the host, usually strengthen the spleen and enhance immunity by increasing the number of beneficial bacteria in the intestinal tract and reducing the number of pathogenic bacteria (Wu et al., 2020; Zhou et al., 2021). With increasing research on biomolecules, several polysaccharides have been reported as prebiotics that fight obesity and improve metabolic diseases continuously (Shi et al., 2020). Baicalin polysaccharides maintain the integrity of the intestinal barrier, and increase the diversity and richness of the intestinal microbiota (Cui et al., 2021). Aloe polysaccharides act as a prebiotic, increasing the number of SCFA-producing bacteria and promoting a range of intestinal functions (Chang Liu et al., 2021). Dietary interventions, probiotic intake, and fecal microbiota transplantation (FMT), which have emerged as potential strategies in the last decade, can be used to reverse gut ecological dysbiosis to homeostasis (Wong and Yu, 2019). Moreover, extracting the polysaccharides from foods or herb medicine can be used to develop prebiotics that regulate and remodel the structure of the intestinal microbiota; this approach appears to be both novel and necessary. In herb medicine and common foods, polysaccharides from the leaves of *Polygonatum sibiricum* regulate the gut microbiota and affect the production of short-chain fatty acids to promote health in mice (Luo et al., 2022b); Seaweed polysaccharides exhibit promising potential for the prevention of obesity and gut microbiota dysbiosis by enriching gut bacterial species with fucoidan-degrading ability (Wei et al., 2022). In mushroom, *Ganoderma lucidum* polysaccharides increase the ratio of *Bacteroides*/*Firmicutes* in the intestinal flora and promote the short chain fatty acids production (Khan et al., 2018); Polysaccharide extracted from *Inonotus obliquus* can modulate gut microbiota by increasing the relative abundance of probiotic bacteria (Hu et al., 2017); Water-insoluble polysaccharide extracted from *P. cocos* can improve hyperglycemia, hyperlipidemia, and hepatic steatosis in mice by regulating the gut microbiota (Sun et al., 2019). The response of the human gut microbiome to bioactive extracts from edible mushrooms represents a study, which deserves our continued attention (Khan et al., 2019; Jianhua Yin et al., 2020; Liu et al., 2021). Consistently, *P. cocos* is an ancient edible and medicinal mushroom, and all three active metabolites exert their different medicinal effects, thereby reducing the relative body weight of mice, affecting the gut microbiota and altering metabolic substances.

Our experimental study found that microbial compositions and structures were not the same following the administration of the three effective substances from *P. cocos* to mice, with PCY demonstrating the strongest probiotic effect. The PCoA results indicated a statistically significant differences between the gut microbiota of the different groups, and samples in the same group were similar. PCX altered the structure of intestinal flora in mice and PCY interestingly reshaped the layout and structure of intestinal microbial colonies; In contrast, the intestinal flora structure of the PCZ group did not change significantly, indicating that PCZ may have a minimal impact on the intestines of mice, and we assume that the triterpenoids in *P. cocos* act on other systems and organs of the living body, such as the cardiovascular system and the immune system; this assumption is consistent with previous studies of triterpenoid saponins (Baosong Chen et al., 2019). The gut microbiota of mice was mainly composed of *Firmicutes* and *Bacteroides*, which accounted for nearly 90% of the total intestinal bacterial community. These species combined with bacteria such as *Aspergillus* and *Clostridium* to form a complete system, which is consistent with the finding of a wide range of previous studies (Meng et al., 2016). At the phylum level, the ratio of *Firmicutes*/Bacteroidetes is considered one of the biological indicators of obesity, and its alteration is closely related to obesity (Ottosson et al., 2018). According to a series of reports, the phyla *Bacillus* and *Firmicutes* contains a large number of glycoside hydrolases that strongly impact the degradation of polysaccharide carbohydrates, which can be fermentatively broken down into various short-chain fatty acids (Turnbaugh et al., 2009). According to published reports, probiotics such as *Lactobacillus* and *Bifidobacterium* can use polysaccharides to produce metabolites, and are strongly associated with obesity (Chen et al., 2018). Probiotics are active microorganisms that are beneficial to the host and maintain intestinal health by modulating host mucosal and systemic immune functions or by regulating the balance of flora in the intestine and promoting nutrient absorption (Chahwan et al., 2019). *Lactobacilli* have potential health benefits at the genus level, including associations with weight loss and improvements in metabolic disorders; accordingly, they have received considerable research in the field of microbiology in the last decade (Han et al., 2021). At the family level, the Lachnospiraceae and Lachnospiraceae-NK4A316 groups are the major butyric acid-producing bacteria that belong to the main flora of *Firmicutes*; this suggests that polysaccharides may promote intestinal health by increasing the proportion of butyric acid producers in the flora to facilitate the synthesis of beneficial metabolites (Louis and Flint, 2017; Ye et al., 2020). The effects of each microbe are different, and different substances cause different impacts on microorganisms. Microbes respond differently to dietary components, owing to different metabolites have various physical properties and pharmacological activities (Zou et al., 2021). In our study, the microbiological analysis data showed that PCY significantly increased the relative abundance of *Lactobacilli*, while PCX conversely decreased the relative abundance of *Lactobacilli*. We speculate that this may be that *P. cocos* water-insoluble polysaccharides are favored by the *Lactobacilli* in the intestine due to its properties. These results further demonstrated that PCY can increase the relative abundance of probiotics, remodel the intestinal biome structure, and optimize the intestinal barrier.

Metabolomic results showed that following administration of the three main metabolites of *P. cocos*, the fecal metabolites of the three groups of mice tested using non-targeted metabolomics showed significantly different profiles, with a greater variation observed in a wide variety of short-chain polypeptide metabolites. The short-chain polypeptides of PCX, PCY, and PCZ significantly increased compared to the CNT group, Miguel et al. found that a metabolic hub consisting of Gly Ser Thr metabolism may influence molecular mechanism related to longevity, which is beneficial to the human body (Aon et al., 2020). Intestinal metabolism is a complex process, non-targeted metabolomics calculates main products of intestinal metabolism, including amino acid, fatty acid, and purine. The metabolite differences in mice treated with the three metabolites of *P. cocos* were mainly in the short-chain polypeptide in present study. However, the intestinal bacterial community mainly breaks down polysaccharides into various short-chain fatty acids (SCFAs) as a source of energy and to regulate intestinal functions, while the concentration of SCFAs may not be detected in the present study method. It may be the reason why PCX and PCZ cause significant differences in intestinal metabolites without altering the structure and composition of the mice gut microbiota. Therefore, we speculate that both PCX, PCY, and PCZ can promote the degradation and digestion of proteins. The correlation analysis between the microbiome and metabolomics revealed that the different metabolites of *P. cocos* caused different intestinal microbial structures, and the differences in microbiomes between the different groups in turn caused differences in metabolites, which is broadly consistent with our conjecture.

Following the treatments with polysaccharides and triterpene saponins, mice resisted pathogen invasion by producing inflammatory cytokines such as IL-10, TNF-α, and IFN-γ, which are key substances of defense against pathogens by macrophages used for activation (Lin et al., 2019). IFN-γ is mainly produced by immune type 1 t helper (Th1) cells to generate non-specific immunity, and IL-10 is an anti-inflammatory and pleiotropic cytokine with important immunomodulatory functions (Hurtubise et al., 2019). IL-10 controls chronic stimulation by gut microbes and food antigens, maintains immune system homeostasis, and is therefore considered one of the most important anti-inflammatory cytokines in humans (Williams et al., 2004). Moreover, it inhibits the secretion of pro-inflammatory cytokines such as TNF-α, IL-10, and IFN-r, inactivates macrophages, reduces the proliferation and differentiation of macrophages, T cells, and B cells, and prevents excessive immune reactions and tissue damage (Qinmei Li et al., 2021). We speculate that the significant increase in IL-10 and TNF-α levels in response to *P. cocos* polysaccharides improved the integrity and function of the intestinal barrier, prevent toxin and bacterial invasion, and reduce the inflammatory responses. *P. cocos* is widely used in the traditional Chinese diet and medicinal culture; as it is usually taken in the form of a water decoction in traditional Chinese medicine to treat diseases; PCX is therefore ingested into the intestinal tract as the main component of *P. cocos* as a therapeutic agent, and the PCY in the residue are usually discarded, which neglects the powerful prebiotic effect of PCY. The above results suggested that we should pay attention to the potential value of PCY which may be developed into a new medicine product.

Although our current study is not exhaustive, that we did not group polysaccharides and triterpenoid saponins at high and low doses in animal experiments, or create animal models with diseases to verify the pharmacological effects of *P. cocos*. In further study, we plan to establish pathological animal models to investigate the mechanism effecting *P. cocos* water-insoluble polysaccharide on the gut microbiota, and apply a targeted metabolomics approach based on SCFAs to explore metabolite differences in deep. To sum up, the results showed that PCY significantly altered the structure of the intestinal microbial community, and both polysaccharides and triterpene saponins caused differences in the composition of metabolites in feces. Specifically, oral administration of PCY can change the structure and abundance of gut microbiota, as well as the composition of intestinal metabolites.

## Conclusion

In summary, our study applied 16S rRNA HTS and a metabolomic approach to determine the effects of three main metabolites of *P. cocos* on mice gut microbiota. We concluded that PCY is the effective bioactive component of *P. cocos* in terms of pharmacological effects on the regulation of gut microbiota in this study. PCY strongly modulate the mice gut microbiota by altering its composition and structure, while PCX and PCZ have caused slight difference in microbiomes. Prominently, PCY significantly increased the number of probiotic bacteria *Lactobacillus* (*p* < 0.01). In addition, the metabolomic results showed the three main active metabolites of *P. cocos* significantly changed the content of short-chain peptides in intestinal metabolites. Collectively, this study has further investigated the pharmacological functions of the three main metabolites from *P. cocos* on regulating gut microbiota. Our findings suggest that PCY is a prominent prebiotic that may be developed into a new prebiotic product; developing this product would help ensure that the potential medicinal value of *P. cocos* can be fully exploited and utilized (Di et al., 2018; Zhu et al., 2018; Nie et al., 2019; Li et al., 2020; Chen et al., 2021; Liang et al., 2021).

## Data Availability

The datasets presented in this study can be found in online repositories. The names of the repository/repositories and accession number(s) can be found below: https://www.ncbi.nlm.nih.gov/bioproject/PRJNA817297.
