# Supplementary material for: Three main metabolites from Wolfiporia cocos (F. A. Wolf) Ryvarden & Gilb regulate the gut microbiota in mice: A comparative study using microbiome-metabolomics
Source: Front Pharmacol. 2022 Aug 3;13:911140. doi: 10.3389/fphar.2022.911140 (PMC9382301; doi:10.3389/fphar.2022.911140)
Supplement: Supplementary file 1 [file DataSheet1.docx]

**Supplementary Materials:**

**Three main metabolites from *Wolfiporia cocos* (F.A. Wolf) Ryvarden & Gilb. regulate the gut microbiota in mice: A comparative study using Microbiome-Metabolomics**

Yong Lai^1,#^, Hailun Yu^1,#^, Huiling Deng^2^, Qi Fang^1^, Hui Lei^1^, Li Liu^1^, Nannan Wu^1^, Xiurong Guo^1,^*, Can Song^1,^*

1. School of Pharmacy, Southwest Medical University, Luzhou, 646000, Sichuan, China

2. Key Laboratory of Condiment Supervision Technology for State Market Regulation, Chongqing Institute for Food and Drug Administration, Chongqing, 401121, China

* Corresponding: cansong@swmu.edu.cn (Can Song), xiurongguo@swmu.edu.cn (Xiurong Guo)

# These authors contributed equally to this work.





Figure S1. Body weight of mice



Figure S2. Food consumption of mice

| **polysaccharides** | **Monosaccharide** |
| --- | --- |
| **PCX** | xylose, mannose, glucose, galactose |
| **PCY** | glucose |

Table S1. Monosaccharide composition of polysaccharides

Table S2. Alpha Diversity Index

|  | **ace** | **shannon** | **simpson** | **shannoneven** | **simpsoneven** | **coverage** |
| --- | --- | --- | --- | --- | --- | --- |
| **PCX**  **PCY**  **PCZ**  **CNT** | 303.375 | 4.49586925 | 0.028374375 | 0.78726125 | 0.13603125 | 1 |
|  | 246.5  297.375  288.125 | 3.9508305  4.164252  4.435118375 | 0.0716615  0.05670725  0.03286475 | 0.7184205  0.737393625  0.78346875 | 0.091950375  0.098787875  0.131646125 | 1  1  1 |


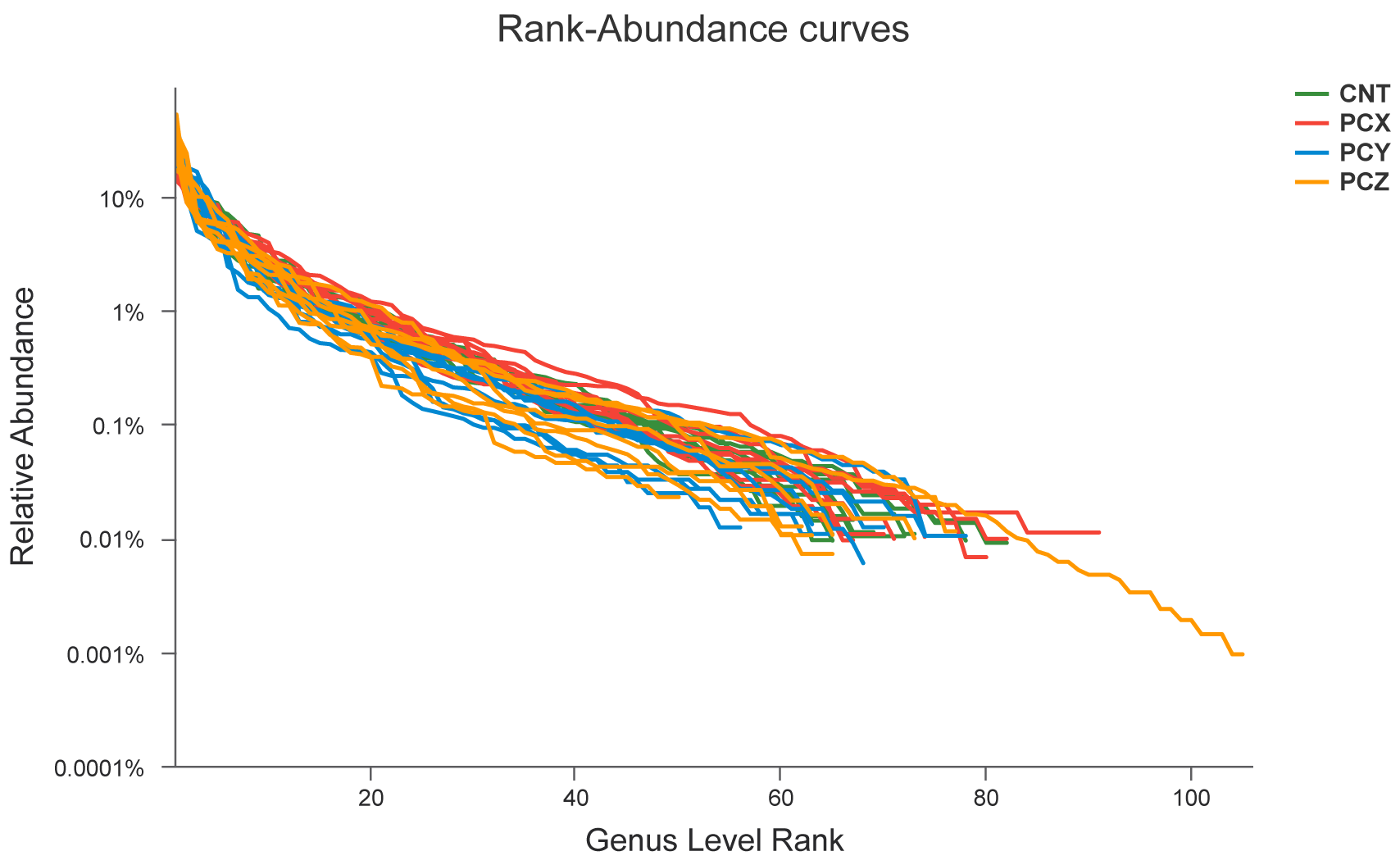
Figure S3. Rank-Abundance curves


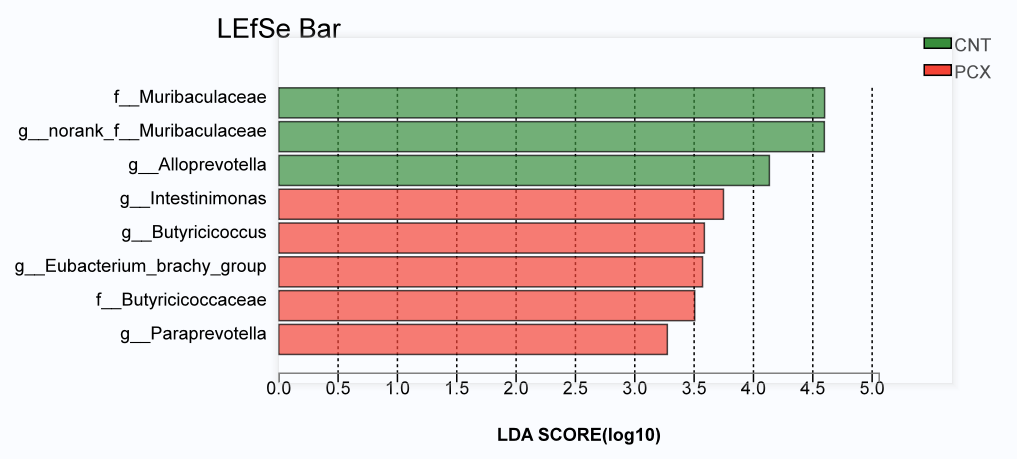


Figure S4. LEfSe Bar between PCX and CNT


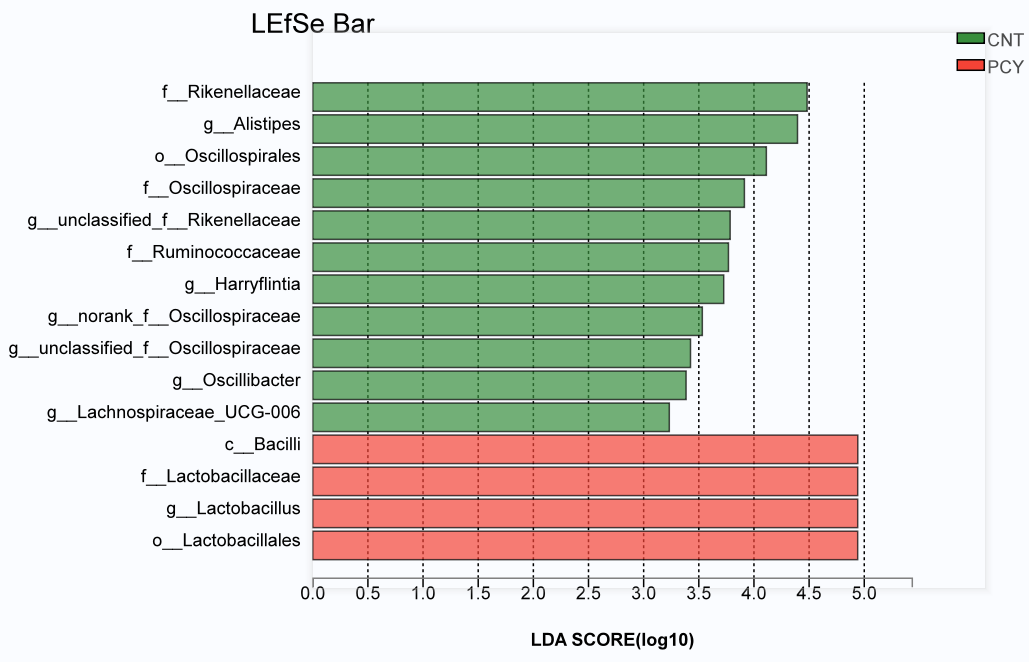


Figure S5. LEfSe Bar between PCY and CNT


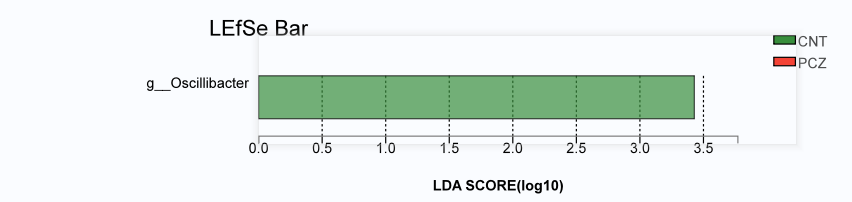


Figure S6. LEfSe Bar between PCZ and CNT
